# Assessment of corneal epithelial thickness mapping by spectral-domain optical coherence tomography

**DOI:** 10.3389/fmed.2024.1459636

**Published:** 2024-09-27

**Authors:** Pedro Tañá-Rivero, Paz Orts-Vila, Pedro Tañá-Sanz, María Ramos-Alzamora, Robert Montés-Micó

**Affiliations:** ^1^Oftalvist Alicante, Alicante, Spain; ^2^Department of Optics and Optometry and Vision Sciences, University of Valencia, Valencia, Spain

**Keywords:** cornea, epithelial thickness, optical coherence tomography, spectral domain, repeatability, reproducibility

## Abstract

**Background:**

To assess corneal epithelial-thickness (ET) mapping resulting from spectral-domain-optical-coherence-tomography (SD-OCT) by analysing its repeatability and reproducibility and its utility for screening corneal-refractive-surgery (CRS) candidates.

**Methods:**

ET was measured in 25-sectors by two-operators. Intra-subject-standard-deviation, coefficient-of-repeatability (CoR) and coefficient-of-variability (CoV) were calculated to evaluate repeatability. Reproducibility was evaluated using a Bland–Altman analysis. Scheimpflug-tomography, refraction, visual acuity, and patient history were used to make a decision on eligibility for CRS. After this decision, the surgeon was shown the patient’s ET map and was asked to reconsider his analysis. The percentage of screenings that changed after evaluating the ET maps was determined.

**Results:**

Forty-three eyes with normal corneas (CRS-group) and 21 eyes not suitable for CRS (non-CRS-group) were studied. For the CRS-group, CoR ranged from 2.03 (central) to 19.73 μm (outer-inferonasal), with the central-sector showing the highest repeatability (CoV: 1.53–1.80%). For the non-CRS-group, CoR ranged from 3.82 (central-middle-superonasal) to 13.42 μm (middle-inferotemporal), with the inner-superonasal-sector showing the highest repeatability (CoV: 2.86–4.46%). There was no statistically significant difference between operators (*p* > 0.01). In the CRS-group, the outcomes showed a narrow 95% limits-of-agreement (LoA) for the central-and inner-nasal-sectors (about 4 μm), and wider for the inner-superior, outer-superotemporal and outer-inferonasal (about 10–14 μm). In the non-CRS-group, they were for the outer superonasal (about 4 μm), and for the middle-inferotemporal and outer-temporal (about 10 μm), respectively. Candidacy for CRS changed in 7.82% of patients after evaluation of the ET maps, with all of them screened-out.

**Conclusion:**

The SD-OCT provided repeatable and reproducible corneal ET measurements and may alter candidacy for CRS.

**Clinical trial registration:**

German Clinical Trials Register: https://drks.de/search/en/trial/DRKS00032797, identifier: DRKS00032797.

## 1 Introduction

The use of spectral-domain optical coherence tomography (SD-OCT), which is based on low coherence interferometry that provides high-resolution, cross-sectional corneal images, enables measurement of corneal epithelial thickness. As a result, and given its increasing popularity in clinical practice, surgeons have gained the ability to frequently measure this parameter and obtain a complete and detailed picture of the corneal structure. Indeed, an accurate corneal epithelial thickness measurement is essential when performing an ocular examination in refractive surgery patients (1). Moreover, its measurement has a wide range of clinical applications, including use as a diagnostic tool for keratoconus and postoperative corneal ectasia (2–5), assessment of ocular surface disorders (6–9), screening for epithelial basement membrane dystrophy (10), the features of limbal stem-cell deficiency (11, 12), or a comparison in normal corneas with different types of astigmatism, for example (13).

One commercially available SD-OCT that can be used to perform corneal epithelial thickness mapping is the Cirrus 5000 HD-OCT (Carl Zeiss Meditec Inc., Jena, Germany). This instrument uses an external cornea-specific lens which, combined with new software, generates automated measurements of corneal epithelial thickness across the cornea. This new software has been developed to be less susceptible to inclusion of the tear-film thickness in the corneal epithelial thickness measurement compared with the previous algorithm (3, 14, 15). This instrument has been shown to be a non-invasive and useful technique for corneal epithelial thickness measurement in normal cornea, post-laser *in situ* keratomileusis (LASIK), corneal disease (i.e., keratoconus), children, dry-eye disease, small incision lenticule extraction (SMILE) and corneal collagen crosslinking (16–21).

During our clinical standard practice, in addition to the patient’s history, refraction, corneal topography, corneal thickness and age, we also use epithelial thickness maps to assess the patient’s candidacy for corneal refractive surgery. However, to the best of our knowledge, no prospective study in adults candidate for refractive surgery assessing the repeatability and reproducibility of this parameter, as measured using the Cirrus 5000 HD-OCT, and the subsequent impact on surgical decision-making, has been published. Consequently, the purpose of the present study was to assess corneal epithelial thickness mapping resulting from SD-OCT by analysing its repeatability and reproducibility and its utility for screening corneal refractive surgery candidates.

## 2 Materials and methods

### 2.1 Study design

This prospective study enrolled consecutive patients proposed for corneal refractive surgery at the Oftalvist center (Alicante, Spain) between October 2023 and March 2024. The study was conducted in accordance with the tenets of the Declaration of Helsinki, with all patients providing written informed consent after receiving an explanation of the purpose of the study before they were enrolled. This study was approved by the Ethics Committee of the Hospital Clínico San Carlos (Madrid, Spain, number 23/591-O_P) and registered at the public German Clinical Trials Registry prior to commencement of the study (identifier: DRKS00032797).

The inclusion criteria were age ≥18 years, written informed consent for participation in the study and data protection, individuals requiring refractive surgery and no active ocular disease. Exclusion criteria were patients who had a history of corneal surgery or any other significant ocular surgery, soft contact lens use in the 2 weeks before measurements, or rigid gas permeable contact lens use in the 4 weeks before measurements, patients who were unable to fixate and follow instructions (inability to comply with the imaging protocol) and those who were pregnant or breastfeeding.

### 2.2 Instrument and measurements

Before being enrolled, all patients underwent a full eye examination, including preoperative corrected distance visual acuity (CDVA), subjective refraction, Pentacam Scheimpflug tomography (Oculus Optikgeräte GmbH, Wetzlar, Germany) and slit-lamp and dilated fundus examinations by ophthalmoscopy.

**Figure 1:**
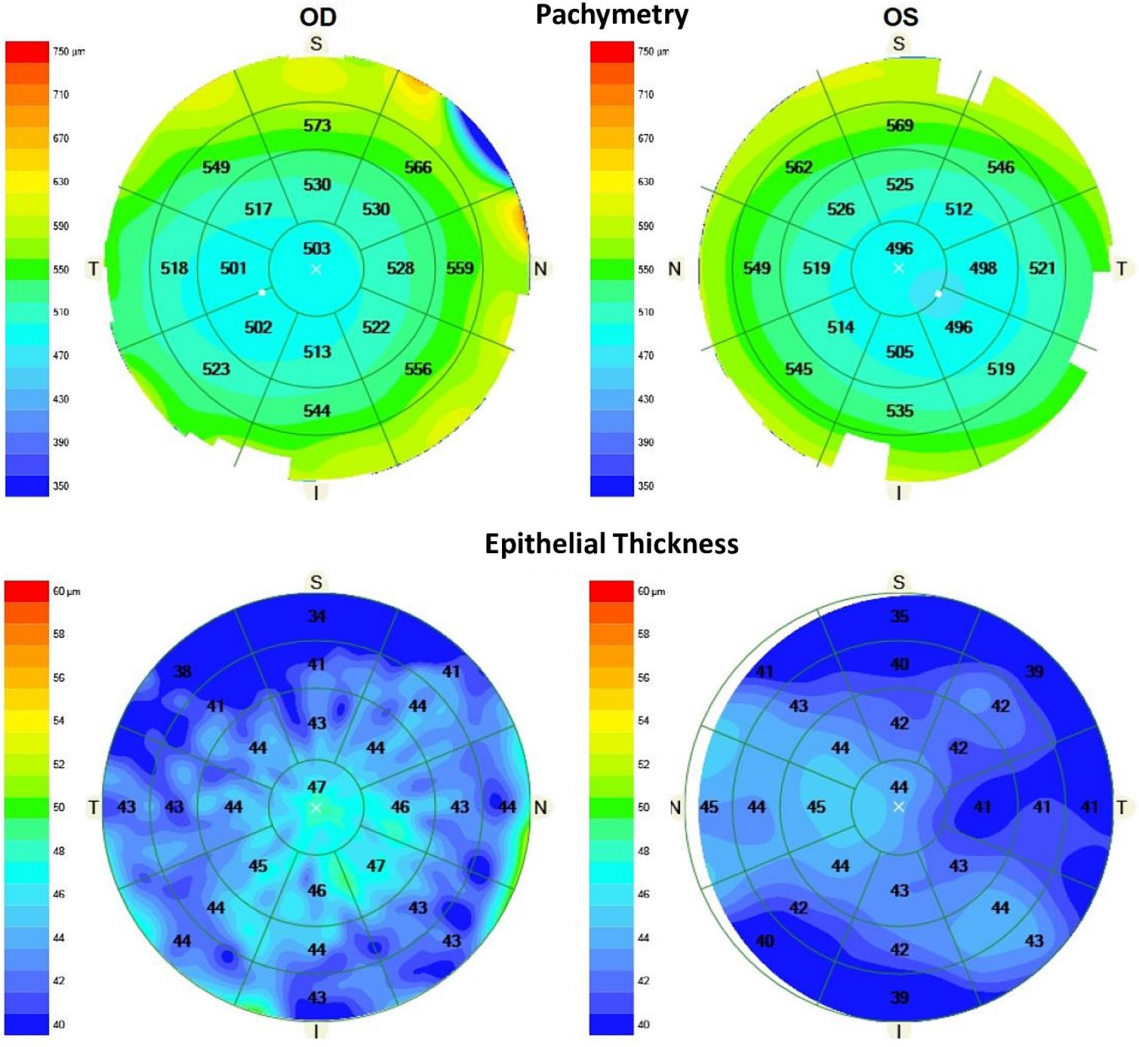
Pachymetry (top) and epithelial thickness (bottom) maps (microns) obtained using the CIRRUS 5000 HD-OCT device showing the mean values for the 25 sectors analysed in a healthy patient for right and left eyes (S, superior; N, nasal; I, inferior; T, temporal).

Specifically, an anterior segment OCT measurement was performed using the Cirrus 5000 HD-OCT. This device operates based on the principle of low-coherence interferometry. Thus, it emits a beam of near-infrared light, which is split into a sample (the cornea in this case) and a reference arm. The interference pattern between the reflected light from these two arms is used to generate detailed cross-sectional images of the cornea. The corneal lens attachment was used to measure the pachymetry and generate the epithelial thickness map (Figure 1). Pachymetry scans do not show signal strength to indicate scan quality. Instead, an image-quality indicator detects whether the scan quality is acceptable. The scan consists of 24 radial B-scan lines (1,024 samples per B-scan) with a scan depth of 2.0 mm. A color-coded thickness map of the corneal was generated after image acquisition. This epithelial thickness map displays the thickness of the outer corneal layer, with “X” marking the vertex for reference. The map is divided into concentric rings with specific diameters of 2 mm for the central ring, 5 mm for the inner ring, 7 mm for the third ring and 9 mm for the outer ring. A total of 25 sectors were analyzed: central, inner nasal, inner superonasal, inner superior, inner superotemporal, inner temporal, inner inferotemporal, inner inferior, inner inferonasal, middle nasal, middle superonasal, middle superior, middle superotemporal, middle temporal, middle inferotemporal, middle inferior, middle inferonasal, outer nasal, outer superonasal, outer superior, outer superotemporal, outer temporal, outer inferotemporal, outer inferior and outer inferonasal. Only one eye per patient (right) was analysed by two skilled operators in a random order. They performed five scans with the Cirrus 5000 HD-OCT in order to assess the repeatability for each operator. Reproducibility was calculated by changing the operator and comparing the results for each of them.

An initial analysis was carried out by the surgeon considering the patient’s age, medical and ophthalmic history, CDVA, refraction, slit-lamp exam and the four-map refractive display from the Pentacam device (axial topography, pachymetry, anterior elevation and posterior elevation, both to the best-fit sphere). The surgeon then approved or rejected the patient’s candidacy for corneal refractive surgery [photorefractive keratectomy (PRK) or LASIK]. After this decision, the surgeon was shown the patient’s epithelial thickness map obtained using the Cirrus 5000 HD-OCT (first measurement of the first operator) and was asked to reconsider this analysis with all the information available. An analysis of a possible change in candidacy was then performed with the following possibilities: “no change in candidacy,” “change and screened-in,” and “change and screened-out.”

### 2.3 Statistical analysis and sample size calculation

All data were analyzed using Microsoft Excel (Microsoft Corp., Redmont, United States), with all data presented as the mean ± standard deviation (SD) and ranges. Repeatability was assessed by calculating the intra-subject standard deviation (S_w_), coefficient of repeatability (CoR) and coefficient of variability (CoV) (22). The CoR was expressed as the SD of the difference between measurements ( $\sqrt{2}\cdot S_{w}$ ). Thus, CoR was calculated as $1.96\sqrt{2}\cdot S_{w}$ and can be approximated as 2.77S_w_. The CoV was calculated as the ratio between S_w_ and the average value: CoV = $S_{w}/x$ . To assess reproducibility, the degree of similarity between the measurements performed by the two different operators was determined by calculating the mean of the five measurements. The paired *t*-test was used to compare the mean values obtained by the two operators. A *p*-value of less than 0.01 was considered statistically significant. In addition, the agreement between these two operators was assessed by performing a Bland–Altman analysis, and the average difference, the confidence interval (CI) of the average difference at 95, and 95% limits of agreement (LoA, calculated as the mean difference ± 1.96 SD) were also calculated.

The required sample size was determined by considering the repeatability for eyes with normal corneas and eyes with corneas not suitable for corneal refractive surgery. Thus, *n* was calculated considering the number of repeated measurements (*m*): $\frac{1.96}{\sqrt{2n\left(m-1\right)}}$ = 0.1 (23). A total of 21 eyes were required, considering a 0.15% confidence in the estimate and five repeated measurements in each group. Since this was a prospective study, and more eyes were expected to be suitable for corneal refractive surgery, all of the suitable eyes from number 21 onwards were also included and analyzed up to a sample of 21 eyes for patients not suitable for refractive surgery.

## 3 Results

In our study, a total of 64 eyes from 64 patients (34 women) were evaluated. The mean age of the patients was 34.08 ± 9.58 years, ranging from 18 to 59 years. The mean spherical equivalent of the eyes evaluated was 3.77 ± 2.99 D, ranging from −13.75 to 4.38 D, and their Snellen decimal CDVA was 0.97 ± 0.08, ranging from 0.55 to 1.00. All eyes were measured by both operators and no problems were encountered during the process. A total of 43 eyes suitable for corneal refractive surgery were considered (refractive surgery group), along with 21 eyes not suitable for corneal refractive surgery (non-refractive group). The repeatability and reproducibility analysis was performed for each of the two groups.

### 3.1 Intraoperator repeatability

The repeatability outcomes for the different sectors analysed in the refractive surgery group eyes by both operators are shown in Table 1 (mean ± SD, S_w_, CoR and CoV parameters). The CoR values ranged from 2.03 (central) to 19.73 μm (outer inferonasal). Measurement of the central sector provided the highest repeatability, with a CoV value of 1.53 and 1.80% for operator 1 and 2, respectively (the lower the CoV, the higher the repeatability). The repeatability outcomes for the different sectors analysed in the non-refractive surgery group eyes are also shown in Table 1. In this case, the CoR values ranged from 3.82 (central middle superonasal) to 13.42 μm (middle inferotemporal). Measurement of the inner superonasal sector provided the highest repeatability, with a CoV value of 2.86 and 4.46% for operator 1 and 2, respectively.

**Table 1:** Intraoperator repeatability outcomes for five epithelial thickness measurements (μm) performed by two operators using the Cirrus 5000 HD-OCT device for the refractive and non-refractive surgery groups.

Sector	Refractive surgery group				Non-refractive surgery group			
	Mean ± SD	S_w_	CoR	CoV (%)	Mean ± SD	S_w_	CoR	CoV (%)
Central								
Operator 1	48.16 ± 3.25	0.73	2.03	1.53	46.86 ± 4.25	1.52	4.23	3.26
Operator 2	48.18 ± 3.16	0.75	2.40	1.80	47.54 ± 4.34	2.23	6.17	4.69
Inner nasal								
Operator 1	46.74 ± 0.61	0.78	2.17	1.68	46.34 ± 3.71	1.40	3.88	3.03
Operator 2	46.85 ± 3.27	0.99	2.75	2.12	47.07 ± 4.54	2.31	6.40	4.91
Inner superonasal								
Operator 1	46.69 ± 2.50	1.58	4.38	3.39	48.41 ± 2.72	1.38	3.83	2.86
Operator 2	46.76 ± 3.19	0.92	2.56	1.98	49.07 ± 3.20	2.18	6.06	4.46
Inner superior								
Operator 1	46.74 ± 4.24	6.16	17.08	13.20	48.51 ± 2.56	1.52	4.22	3.14
Operator 2	46.24 ± 3.34	1.04	2.90	2.27	48.97 ± 3.26	2.01	5.57	4.11
Inner superotemporal								
Operator 1	45.98 ± 3.25	1.41	3.91	3.07	48.10 ± 2.81	1.42	3.98	2.96
Operator 2	45.91 ± 3.26	1.05	2.93	2.31	48.65 ± 3.36	2.25	6.24	4.63
Inner temporal								
Operator 1	46.32 ± 3.23	0.96	2.68	2.09	46.62 ± 3.34	1.48	4.11	3.19
Operator 2	46.29 ± 3.16	1.13	3.13	2.45	47.21 ± 3.51	1.98	5.49	4.20
Inner inferotemporal								
Operator 1	46.99 ± 3.06	0.85	2.36	1.82	45.62 ± 4.98	1.45	4.02	3.19
Operator 2	47.00 ± 3.01	1.37	3.80	2.92	46.17 ± 4.97	2.38	6.59	5.16
Inner inferior								
Operator 1	48.04 ± 3.20	0.78	2.18	1.64	46.24 ± 4.52	1.61	4.47	3.50
Operator 2	48.13 ± 3.21	1.50	4.15	3.12	46.77 ± 4.72	2.50	6.93	5.35
Inner inferonasal								
Operator 1	47.82 ± 3.30	0.74	2.07	1.57	45.87 ± 4.83	1.56	4.33	3.42
Operator 2	47.97 ± 3.16	1.16	3.22	2.43	46.31 ± 5.26	2.59	7.19	5.60
Middle nasal								
Operator 1	45.50 ± 3.20	1.23	3.42	2.72	46.01 ± 3.89	1.50	4.16	3.27
Operator 2	45.73 ± 3.26	1.15	3.18	2.52	46.48 ± 4.42	5.97	6.76	5.26
Middle superonasal								
Operator 1	46.01 ± 2.99	1.07	2.98	2.34	47.13 ± 2.33	1.37	3.82	2.93
Operator 2	46.15 ± 3.22	0.99	2.75	2.15	47.56 ± 2.71	1.98	5.50	4.18
Middle superior								
Operator 1	44.38 ± 2.93	1.62	4.50	3.67	45.79 ± 2.05	1.76	4.89	3.86
Operator 2	44.12 ± 3.36	1.74	4.82	3.95	46.01 ± 2.62	2.00	5.55	4.36
Middle superotemporal								
Operator 1	44.13 ± 2.92	1.17	3.24	2.65	46.56 ± 3.02	1.56	4.33	3.36
Operator 2	43.98 ± 3.24	1.54	4.26	3.50	46.90 ± 3.40	2.05	5.69	4.38
Middle temporal								
Operator 1	45.65 ± 2.72	1.08	3.00	2.38	47.19 ± 3.11	1.46	4.04	3.10
Operator 2	45.59 ± 2.94	1.12	3.10	2.46	48.00 ± 3.86	2.18	6.04	4.54
Middle inferotemporal								
Operator 1	46.01 ± 2.86	1.69	3.60	2.83	46.83 ± 3.74	1.78	4.95	3.82
Operator 2	46.06 ± 2.94	1.43	3.98	3.12	47.67 ± 4.45	4.84	13.42	10.17
Middle inferior								
Operator 1	47.23 ± 3.56	2.98	8.26	6.32	48.25 ± 4.54	1.95	5.40	4.05
Operator 2	47.50 ± 3.73	1.59	4.41	3.36	48.70 ± 4.98	2.78	7.70	5.71
Middle inferonasal								
Operator 1	47.13 ± 3.51	1.52	4.21	3.23	47.40 ± 5.39	2.51	6.96	5.30
Operator 2	47.24 ± 3.48	1.69	4.70	3.59	47.32 ± 5.68	2.98	8.27	6.31
Outer nasal								
Operator 1	45.77 ± 3.24	2.77	7.68	6.07	46.50 ± 3.94	2.03	5.63	4.38
Operator 2	46.07 ± 3.49	1.55	4.31	3.38	46.49 ± 3.65	2.07	5.74	4.46
Outer superonasal								
Operator 1	44.93 ± 3.17	1.32	3.66	2.95	44.85 ± 2.41	1.51	4.19	3.37
Operator 2	45.07 ± 3.10	1.25	3.46	2.78	45.23 ± 2.76	1.75	4.86	3.88
Outer superior								
Operator 1	42.28 ± 3.93	1.52	4.22	3.60	43.06 ± 2.88	2.36	6.54	5.49
Operator 2	42.03 ± 3.80	1.83	5.08	4.37	43.03 ± 3.62	1.96	5.44	4.57
Outer superotemporal								
Operator 1	43.02 ± 3.09	2.38	6.60	5.54	44.73 ± 2.57	2.99	8.30	6.70
Operator 2	43.35 ± 3.47	4.03	11.16	9.30	45.22 ± 3.38	2.72	7.55	6.03
Outer temporal								
Operator 1	45.96 ± 2.77	1.37	3.79	2.98	47.70 ± 3.45	1.83	5.08	3.85
Operator 2	45.83 ± 2.88	1.55	4.230	3.39	48.70 ± 4.83	4.49	12.46	9.24
Outer inferotemporal								
Operator 1	46.65 ± 3.19	3.14	8.69	6.73	47.11 ± 4.03	2.67	7.41	5.68
Operator 2	46.77 ± 3.14	4.27	11.84	9.15	47.45 ± 3.87	2.92	8.10	6.17
Outer inferior								
Operator 1	46.91 ± 3.67	1.32	3.67	2.83	47.56 ± 3.94	2.21	6.13	4.66
Operator 2	46.81 ± 3.78	1.75	4.85	3.74	47.99 ± 4.55	3.91	10.85	8.16
Outer inferonasal								
Operator 1	47.67 ± 3.64	4.75	6.04	4.58	47.85 ± 3.89	2.25	6.23	4.71
Operator 2	48.32 ± 4.86	7.12	19.73	14.74	48.24 ± 4.07	2.25	6.24	4.67

SD, standard deviation; S_w_, intra-subject standard deviation; CoR, coefficient of repeatability; CoV, coefficient of variability.

### 3.2 Interoperator reproducibility

**Figure 2:**
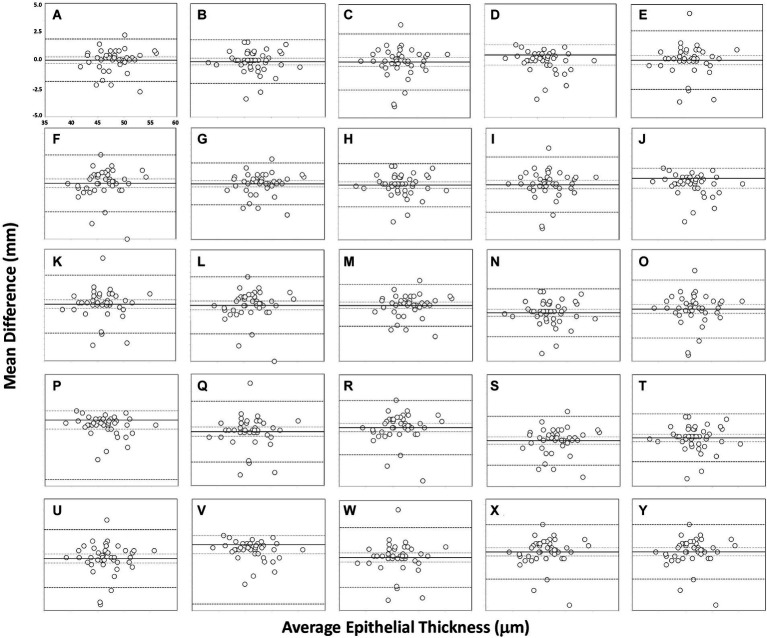
Bland–Altman plots showing agreement in measurements for different sectors for the two operators in the refractive surgery group: central **(A)**, inner nasal **(B)**, inner superonasal **(C)**, inner superior **(D)**, inner superotemporal **(E)**, inner temporal **(F)**, inner inferotemporal **(G)**, inner inferior **(H)**, inner inferonasal **(I)**, middle nasal **(J)**, middle superonasal **(K)**, middle superior **(L)**, middle superotemporal **(M)**, middle temporal **(N)**, middle inferotemporal **(O)**, middle inferior **(P)**, middle inferonasal **(Q)**, outer nasal **(R)**, outer superonasal **(S)**, outer superior **(T)**, outer superotemporal **(U)**, outer temporal **(V)**, outer inferotemporal **(W)**, outer inferior **(X)** and outer inferonasal **(Y)**. The mean (continuous line), lower and upper limits of agreement [±1.96 SD (standard deviation), peripheral dotted lines], and the lower and upper confidence intervals (95%) are depicted.

**Figure 3:**
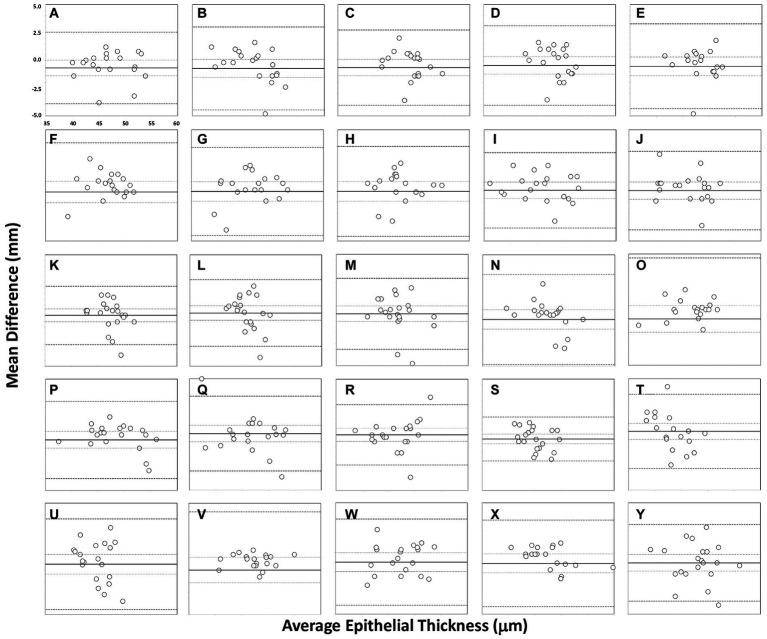
Bland–Altman plots showing agreement in measurements for different sectors for the two operators in the non-refractive surgery group: central **(A)**, inner nasal **(B)**, inner superonasal **(C)**, inner superior **(D)**, inner superotemporal **(E)**, inner temporal **(F)**, inner inferotemporal **(G)**, inner inferior **(H)**, inner inferonasal **(I)**, middle nasal **(J)**, middle superonasal **(K)**, middle superior **(L)**, middle superotemporal **(M)**, middle temporal **(N)**, middle inferotemporal **(O)**, middle inferior **(P)**, middle inferonasal **(Q)**, outer nasal **(R)**, outer superonasal **(S)**, outer superior **(T)**, outer superotemporal **(U)**, outer temporal **(V)**, outer inferotemporal **(W)**, outer inferior **(X)** and outer inferonasal **(Y)**. The mean (continuous line), lower and upper limits of agreement [±1.96 SD (standard deviation), peripheral dotted lines], and the lower and upper confidence intervals (95%) are depicted.

The reproducibility outcomes for the different sectors analysed for the refractive and non-refractive surgery groups are shown in Table 2. This table shows the mean difference ± SD, 95% CI, 95% LoA, and LoA width for the different sectors analysed. There was no statistically significant difference between the values obtained by the two operators (*p* > 0.01) for either the refractive or the non-refractive surgery group. The outcomes for the agreement between the two operators for the different sectors are shown in Figures 2, 3 for the refractive and non-refractive surgery groups, respectively. The Bland–Altman plots in Figure 2 show a narrow 95% LoA for the central and inner nasal sectors (about 4 μm) and a wider 95% LoA for the inner superior, outer superotemporal and outer inferonasal sectors (about 10–14 μm). Similarly, the Bland–Altman plots in Figure 3 show a narrow 95% LoA for the outer superonasal sector (about 4 μm) and a wider 95% LoA for the middle inferotemporal and outer temporal sectors (about 10 μm).

**Table 2:** Mean differences (μm) between the two operators when using the Cirrus 5000 HD-OCT device for the refractive and non-refractive surgery groups.

Sector	Refractive surgery group					Non-refractive surgery group				
	Mean difference ± SD	95% CI	95% LoA	LoA width	*p*-value	Mean difference ± SD	95% CI	95% LoA	LoA Width	*p*-value
Central	−0.018 ± 0.951	−0.303, 0.266	−1.883, 1.845	3.728	0.449	−0.685 ± 1.642	−1.388, 0.017	−3.905, 2.534	6.439	0.035
Inner nasal	−0.107 ± 0.985	−0.401, 0.187	−2.038, 1.824	3.861	0.240	−0.731 ± 1.902	−1.544, 0.038	−4.459, 2.997	7.456	0.047
Inner superonasal	−0.069 ± 1.260	−0.446, 0.307	−2.540, 2.400	4.940	0.359	−0.652 ± 1.725	−1.390, 0.085	−4.033, 2.729	6.762	0.049
Inner superior	−0.502 ± 2.989	−0.391, 1.396	−5.358, 6.363	11.721	0.138	−0.461 ± 1.822	−1.241, 0.318	−4.034, 3.110	7.144	0.130
Inner superotemporal	0.081 ± 1.131	−0.313, 0.475	−2.502, 2.664	5.166	0.346	−0.545 ± 1.945	−1.377, 0.287	−4.358, 3.267	7.265	0.107
Inner temporal	0.023 ± 1.308	−0.368, 0.414	−2.540, 2.587	5.127	0.454	−0.590 ± 2.254	−1.555, 0.374	−5.009, 3.828	8.837	0.122
Inner inferotemporal	−0.018 ± 1.282	−0.402, 0.365	−2.531, 2.494	5.025	0.462	−0.547 ± 2.020	−1.412, 0.317	−4.508, 3.413	7.922	0.114
Inner inferior	−0.088 ± 1.248	−0.491, 0.315	−2.731, 2.554	5.285	0.335	−0.531 ± 2.060	−1.412, 0.350	−4.569, 3.507	8.076	0.126
Inner inferonasal	−0.148 ± 1.146	−0.492, 0.194	−2.396, 2.098	4.494	0.200	−0.440 ± 1.729	−1.180, 0.299	−3.829, 2.948	6.779	0.128
Middle nasal	−0.232 ± 1.360	−0.639, 0.174	−2.899, 2.434	5.334	0.134	−0.466 ± 1.802	−1.238, 0.304	−4.000, 4.066	7.066	0.125
Middle superonasal	−0.143 ± 1.162	−0.490, 0.204	−2.421, 2.135	4.556	0.212	−0.431 ± 1.338	−1.003, 0.141	−3.054, 2.192	5.246	0.078
Middle superior	0.252 ± 1.498	−0.195, 0.701	−2.685, 3.190	5.875	0.138	−0.220 ± 1.529	−0.875, 0.434	−3.219, 2.778	5.997	0.258
Middle superotemporal	0.154 ± 1.240	−0.216, 0.525	−2.277, 2.586	4.863	0.209	−0.340 ± 1.631	−1.038, 0.357	−3.539, 2.858	6.396	0.175
Middle temporal	0.060 ± 1.230	−0.307, 0.428	−2.351, 2.472	4.822	0.374	−0.811 ± 2.061	−1.694, 0.070	−4.853, 3.229	8.082	0.043
Middle inferotemporal	−0.046 ± 1.406	−0.467, 0.374	−2.802, 2.709	5.512	0.415	−0.840 ± 2.801	−2.039, 0.358	−6.331, 4.650	10.982	0.092
Middle inferior	−0.269 ± 1.866	−0.828, 0.288	−3.928, 3.388	7.316	0.174	−0.447 ± 1.766	−1.203, 0.308	−3.909, 3.014	6.924	0.130
Middle inferonasal	−0.102 ± 1.385	−0.516, 0.312	−2.817, 2.613	5.430	0.315	0.073 ± 1.716	−0.660, 0.808	−3.291, 3.438	6.729	0.423
Outer nasal	−0.307 ± 1.863	−0.864, 0.250	−3.959, 3.345	7.303	0.143	0.007 ± 1.385	−0.585, 0.599	−2.705, 2.719	5.423	0.491
Outer superonasal	−0.137 ± 1.288	−0.523, 0.247	−2.662, 2.387	5.049	0.246	−0.376 ± 1.012	−0.809, 0.057	−2.361, 1.609	3.970	0.052
Outer superior	−0.177 ± 1.701	−0.687, 0.338	−3.513, 3.157	6.671	0.264	0.270 ± 1.695	−0.455, 0.996	−3.053, 3.594	6.647	0.254
Outer superotemporal	−0.330 ± 2.623	−1.114, 0.454	−5.473, 4.183	10.286	0.207	−0.484 ± 2.078	−1.374, 0.404	−4.558, 3.589	8.147	0.149
Outer temporal	0.130 ± 1.327	−0.266, 0.527	−2.471, 2.732	5.203	0.262	−0.995 ± 2.671	−2.138, 0.147	−6.231, 4.241	10.473	0.052
Outer inferotemporal	−0.114 ± 2.433	−0.841, 0.613	−4.884, 4.656	9.540	0.380	−0.333 ± 1.974	−1.178, 0.511	−4.204, 3.537	7.741	0.224
Outer inferior	0.097 ± 1.200	−0.261, 0.456	−2.255, 2.450	4.704	0.298	−0.435 ± 1.983	−1.284, 0.413	−4.323, 3.451	7.774	0.163
Outer inferonasal	−0.646 ± 3.632	−1.732, 0.439	−7.765, 6.472	14.238	0.125	−0.388 ± 1.756	−1.139, 0.363	−3.831, 3.055	6.886	0.162

SD, standard deviation; CI, confidence interval; LoA, limit of agreement.

### 3.3 Changes in candidacy

The candidacy for corneal refractive surgery did not change in 92.18% of eyes (*n* = 59) and changed in 7.82% of eyes (*n* = 5) after evaluation of their epithelial thickness maps, with all of these eyes screened-out (*n* = 5). This means that 5 of 64 eyes that were initially considered suitable for corneal refractive surgery based on standard preoperative evaluations alone were subsequently considered not suitable for corneal refractive surgery after the surgeon had analysed the epithelial thickness measurements obtained (due to irregularities and/or low values). The recommended surgical option after being deemed not suitable for corneal refractive surgery was the implantation of a phakic intraocular lens.

## 4 Discussion

Measurement of the epithelial thickness has been shown to be an excellent screening tool for corneal diseases (2–5), amongst other applications (6–13). Specifically, corneal refractive surgery requires the most accurate and precise corneal measurements possible, and an epithelial thickness map can be used as an excellent tool to assess a patient’s candidacy for this surgery. Several SD-OCT devices are currently available on the market for this purpose. The Cirrus 5000 HD-OCT, for example, provides epithelial thickness measurements derived from the corneal SD-OCT images obtained. To the best of our knowledge, this is the first prospective study to assess the repeatability and reproducibility of this parameter measured with the Cirrus 5000 HD-OCT and the impact thereof on surgical decision-making.

The superior corneal epithelium has been shown to be significantly thinner than inferior areas in normal eyes (2, 24), possibly as a result of the friction resulting from the mechanical dynamics of blinking (25). Our results showed excellent repeatability and reproducibility in all the different sectors measured for both refractive and non-refractive surgery eyes. It should be noted, however, that is difficult to directly compare our results with those obtained using other devices due to the different area of the sectors analyzed. However, it is worth mentioning some of them. For example, Ge et al. (26) used four OCT devices (two prototypes, the RTVue Optovue and the Visante) and found CoR values of less than 2.3 μm in healthy subjects and 4.9 μm in LASIK patients in the central epithelial thickness. Our results are in broad agreement, with values of 2.03–4.40 μm and 4.23–6.17 μm for the refractive and non-refractive surgery groups, respectively (see Table 1). In another study, Prakash et al. (27) used the Cirrus HD-OCT in 210 healthy eyes and obtained a CoV for the corneal epithelial thickness reliability of 2.3%, and Georgeon et al. (28) reported a mean CoV of less than 6% using the MS39 and Optovue RTVue-100 devices in healthy eyes. Similarly, Feng et al. (29) assessed the repeatability of the epithelial thickness in virgin, post-laser refractive surgery and keratoconic eyes using the Anterion SS-OCT and the Avanti SD-OCT, obtaining S_w_ values ranging from 0.60 to 1.36 μm and from 0.75 to 1.96 μm, respectively. Recently, Sikorski (30) obtained CoV values ranging from 1.12% (superior) to 2.88% (superior-temporal_out) in 40 healthy eyes using the REVO NX device. Our results are shown in Table 1 for the two groups, operators and different sectors assessed for comparative purposes. For reproducibility, our LoA widths were not clinically significant for any sector measured, with a maximum of 10–14 μm for both groups of eyes examined (see Table 2 and Figures 2, 3). Other authors, such as Prakash et al. (27), obtained a CoV value for reproducibility of 3.5%, and Sikorski (30) reported CoV values ranging from 1.40% (central) to 3.37% (superior-nasal_out).

Various studies in the literature using the same instrument, mainly of a retrospective nature, have analyzed the measurement of corneal epithelial thickness for different applications (16–21). For example, in a retrospective study, Sha et al. (16) assessed the repeatability and reproducibility of the Cirrus 5000 HD-OCT in one eye for 137 patients divided into three groups: normal cornea (*n* = 45), post-LASIK (*n* = 40) and corneal pathology (*n* = 37). These authors analyzed 25 sectors, with three measurements being taken on three devices by three operators. Their results revealed that the CoV was better than 6% for all sectors in normal corneas, with a reproducibility limit that would allow detection of a change of 3–7 μm. These values were 7.5 and 6.5%, and 3–9 μm and 5–6 μm, for post-LASIK and corneal pathology, respectively. They concluded that this instrument exhibits clinically acceptable repeatability and reproducibility limits and can be used to detect patients with keratoconus. Our results (see Tables 1, 2 for detailed values) showed better outcomes for CoV in the central sector (less than 2%) for the refractive surgery group (normal cornea), with higher percentages than these authors except for some sectors, such as inner superior and outer inferonasal. The percentages for the non-refractive surgery group were, in general, less than 5–6%, except for middle inferotemporal (about 10%). In a similar study, Baghdasaryan et al. (17) analyzed 36 healthy eyes from 18 individuals (10 male) using the Cirrus 5000 HD-OCT in order to obtain a distribution profile of the corneal epithelial thickness, and its related characteristics, for both males and females. They reported corneal epithelial characteristics in healthy young subjects over a larger corneal area, and showed analysis of the peripheral corneal epithelium to be useful in disorders or therapies that specifically affect these regions (i.e., peripheral corneal degenerations). Specifically, they provided mean values for four concentric ring-shaped zones (0–2, 2–5, 5–7 and 7–9 mm) centered on the center of the cornea, and also between superior and inferior portions of the cornea within the 2–5 mm and 5–7 mm rings (superior, inferior, temporal, nasal, superonasal, inferotemporal, superotemporal and inferionasal). This article was the first to report data beyond the 6 mm diameter area, which is important in some clinical corneal alterations. Specifically, they found that the corneal epithelial thickness is thinner in the periphery and is essentially unaffected by sex, although there is a trend toward a thinner superior in males. Similarly, Loureiro et al. (18) assessed 60 eyes from 60 healthy children (aged between 8 and 18 years) using the Cirrus 5000 HD-OCT instrument to establish the first normative Caucasian database for this age group. They analyzed 25 sectors and reported that the average corneal epithelial thickness was lower in the peripheral zones and thinner in the superior area than in the inferior (*p* < 0.05). They also showed that it was thicker in boys than in girls (*p* < 0.05) and proposed that this parameter may be influenced by sex. Our results agree with both studies since we found mean values ranging from 42–43 μm (outer superior and outer superotemporal) to 48 μm (central) in the refractive surgery group (normal cornea). It should be noted that our sample contained 53.12% women with a mean age of 34.08 ± 9.58 years, ranging from 18 to 59 years. In a specific study related to dry eye, Loureiro et al. (19) specifically measured the changes in corneal epithelial thickness before and after topical treatment in primary Sjögren syndrome-associated dry eye disease. Thus 40 female eyes (20 with this disease and 20 controls) were evaluated after 4 weeks of treatment with preservative free 1 mg/mL sodium hyaluronate. These authors found that corneal epithelial thickness was thinner in the superior area in Sjögren syndrome-associated dry eye disease and, additionally, the four-week treatment with artificial tears thickened the superior epithelium and improved tear film measurements.

In a retrospective study, Kim et al. (20) evaluated the changes in corneal epithelium thickness induced by combined SMILE and accelerated corneal collagen crosslinking (SMILE-xtra) for myopia compared with standard SIME surgery. They analyzed 31 and 36 eyes for SMILE-xtra and SMILE, respectively, and measured 17 zones within the central 7 mm zone at preoperative and 1, 3, and 6 months, postoperative. Both groups showed the greatest increase in corneal epithelium thickness in the paracentral area and inferotemporal area, respectively, at 6 months. They concluded that, despite the relatively large corneal ablation with SMILE-xtra, there was no significant difference in the corneal epithelial remodeling pattern compared to the SMILE group. Salman et al. (21) aimed to investigate the diagnostic ability of corneal epithelial thickness measurement in differentiating between keratoconus, suspected keratoconus and normal corneas. These authors retrospectively analyzed 144 eyes separated into these three groups based on the Sirius device classification software and used the areas under the receiver operator characteristic (ROC) curve to perform an inter-group comparison and determine the discrimination capacity of the Cirrus 5000 HD-OCT instrument. The ROC curve analysis revealed an excellent predictive ability for the epithelial thickness variables minimum (0–2 mm), minimum-maximum (0–2 mm), superonasal-inferotemporal (2–5 mm), minimum-maximum (2–5 mm), and minimum (2–5 mm) to detect keratoconus (area under the curve >0.9, all). However, epithelial thickness variables were not strong enough (area under the curve <0.8, all) to differentiate between suspected keratoconus and normal eyes, with the highest diagnostic power for minimum-maximum (2–5 mm; AUC = 0.71). They concluded that epithelial thickness measurements can be used to differentiate between keratoconus and normal eyes, with a high level of certainty, with minimum-maximum (2–5 mm) being the parameter best suited to detecting suspected keratoconus.

Our results showed that candidacy for corneal refractive surgery did not change in 92.18% of eyes but changed in 7.82% of eyes after evaluation of their epithelial thickness maps, with all of these eyes screened-out. In this sense, in relation to the utility of corneal epithelial thickness measurements, in a similar study Asroui et al. (1) determined the impact of corneal epithelial thickness maps on screening for refractive surgery candidacy. These authors used the Avanti RTVue XR OCT in 100 consecutive patients in conjunction with the information provided by the Pentacam HR. Their results revealed that candidacy for corneal refractive surgery changed in 16% of patients after evaluation of this map, with 10% of patients screened-in and 6% screened-out. They also noted that the surgery of choice changed for 16% of patients, and the ranking of surgical procedures from most to least favorable changed for 25% of patients. Specifically, they found that a total of 11% of patients gained eligibility for LASIK, whereas 8% lost eligibility for LASIK. They also concluded that epithelial thickness mapping obtained by OCT altered candidacy for corneal refractive surgery, as well as choice of surgery, in a substantial percentage of patients, and was therefore a valuable tool for screening evaluations. Finally, they found that the use of this map resulted in screening in a slightly higher percentage of patients for corneal refractive surgery. As indicated previously, our results, reveal that candidacy for corneal refractive surgery change in 7.82% of eyes.

Our study show that analysis of the epithelial thickness map changed the candidacy for corneal refractive surgery in our cohort, where about 8% of the eyes were excluded. This leads us to conclude that the mapping of this parameter may alter the surgical decision-making, thereby changing our daily clinical practice and benefiting patients for their most adequate refractive surgery procedure. We consider that, despite being subjective, this measurement should be incorporated into the preoperative assessment in order to provide surgeons with more information in order to make a final decision on candidacy for this procedure.

Our study has some limitations. The first is that, despite calculating the sample size for repeatability and reproducibility, our sample is limited, specifically for those eyes not suitable for corneal refractive surgery. Second, in relation to its utility for refractive surgery assessment, the nature of the decision for candidacy is subjective and cannot be extrapolated to other surgeons with different clinical experience. We should indicate that the surgeon in this study has several years of experience using these maps for corneal refractive surgery. In addition, we fully agree with Asrouri et al. (1), who noted that the impact of decision changes on actual clinical outcomes cannot be ascertained. As such, a further study with a larger sample, more surgeons with different clinical experience, and long-term follow-up of the cohort of patients candidate for corneal refractive surgery, and those who are not, is required to support these findings.

In conclusion, this study has two main implications for surgeons and clinicians involved in the management of refractive surgery patients. First, the outcomes of our study reveal that the repeatability and reproducibility of epithelial thickness measurements provided by spectral-domain OCT are good and the limits of the different metrics obtained are clinically acceptable. Second, the measurement of epithelial thickness may be useful for detecting patients with corneal abnormalities and may alter candidacy for corneal refractive surgery. As such, epithelial thickness mapping should be carried out systematically before a refractive surgery procedure.

Competing interests.

P. Tañá-Rivero declares grants for studies from Alcon Healthcare SA, AST Products Inc., BVI Inc., Carl Zeiss, Hoya Surgical AG, Humanoptics Holding AG, Johnson & Johnson Surgical Vision, Staar Surgical AG and Vialase Inc. Paz Orts-Vila declares grants for studies for Alcon Laboratories, AST Products, BVI, Carl Zeiss Meditec, Hoya, HumanOptics and Johnson&Johnson. Robert Montés-Micó declares research and consulting contracts with BVI Inc. and STAAR Surgical AG through the University of Valencia. The authors have no financial or proprietary interest in any product, method or material described herein.

## Data Availability

The original contributions presented in the study are included in the article/supplementary material, further inquiries can be directed to the corresponding author/s.
